# Competition for space during bacterial colonization of a surface

**DOI:** 10.1098/rsif.2015.0608

**Published:** 2015-09-06

**Authors:** Diarmuid P. Lloyd, Rosalind J. Allen

**Affiliations:** SUPA, School of Physics and Astronomy, University of Edinburgh, James Clerk Maxwell Building, Peter Guthrie Tait Road, Edinburgh EH9 3FD, UK

**Keywords:** ecological competition, spatial population expansion, spatial patterning, multicellular self-assembly, microscopy, computer simulations

## Abstract

Competition for space is ubiquitous in the ecology of both microorganisms and macro-organisms. We introduce a bacterial model system in which the factors influencing competition for space during colonization of an initially empty habitat can be tracked directly. Using fluorescence microscopy, we follow the fate of individual *Escherichia coli* bacterial cell lineages as they undergo expansion competition (the race to be the first to colonize a previously empty territory), and as they later compete at boundaries between clonal territories. Our experiments are complemented by computer simulations of a lattice-based model. We find that both expansion competition, manifested as differences in individual cell lag times, and boundary competition, manifested as effects of neighbour cell geometry, can play a role in colonization success, particularly when lineages expand exponentially. This work provides a baseline for investigating how ecological interactions affect colonization of space by bacterial populations, and highlights the potential of bacterial model systems for the testing and development of ecological theory.

## 1. Introduction

Spatial structure can have profound effects on the composition and dynamics of ecological communities. Ecological theory suggests that even in spatially uniform habitats, local neighbour interactions can lead to a variety of phenomena including competitor coexistence owing to trade-offs between life-history strategies (e.g. between local competitive ability and dispersal) [1,2], community self-organization [3], travelling waves of species dominance [4,5] and spatial variations in the prevalence of parasites or other traits [6,7]. Spatial variations in habitat type can produce even more varied ecological outcomes [8]. However, testing such predictions with well-controlled experiments on spatially structured populations is often challenging (although not impossible [9]) for ecosystems consisting of macro-organisms, i.e. animals or plants.

Laboratory studies with microorganisms provide a way to test ecological theory that can overcome many of the practical issues associated with working with macro-organisms. While a large body of work exists for protist ecosystems [10,11], the potential of bacterial populations for testing ecological and evolutionary theory has only recently become widely recognized [12], as has the importance of using such theory to understand bacterial communities in the natural environment [13]. For bacterial populations, key studies have demonstrated the importance of spatial structure in promoting competitive restraint [14], maintaining coexistence in systems with cyclic dominance [15], favouring the evolution of toxins [16] and speeding up the evolution of antibiotic resistance [17–19]. Of particular interest are recent studies of range expansion, in which patterns of clonal dominance are visualized using fluorescently labelled strains of bacteria or yeast, as a population expands across the empty surface of an agar plate [20–22]. This work has shown that neutral fluctuations at the tips of expanding populations can have strong effects on genetic diversity [23,24]. These experiments provide a powerful tool for the testing and development of theory, because fitness differences between strains can be measured with high accuracy, the spatial fates of different lineages can be tracked in detail, and the physical and ecological interactions between individual organisms are relatively simple [25–31]. The spatial patterns formed by expanding and competing bacterial lineages are also of interest in a synthetic biology context, where the aim is to produce the controlled local patterns of differential gene expression [32].

In this paper, we use an experimental bacterial model system, combined with computer simulations, to address a different ecological scenario: the colonization of an empty terrain by initially scattered individuals that proliferate to occupy contiguous patches, competing for space at the patch boundaries. This scenario is common in ecology; examples are to be found among lichens [33], algae [34], liverworts [35], vascular plants [9] and territorial animals [36]. In such a situation, where organisms compete locally for space (or, more generally, for resources), ecological theory points to several different processes that are at play [2,36,37]: expansion competition, in which a species captures space not already occupied by another, lottery competition, in which individuals compete to occupy space released when another dies, and overgrowth, or the capturing of space by direct competition at boundaries between species. For a particular ecosystem, a key task is to determine which of these mechanisms plays the major role. More generally, one would like to predict how the various mechanisms involved influence the spatial patterns of genetic mixing which emerge.

In our experiments, two fluorescently labelled strains of *Escherichia coli* bacteria compete locally for space, starting from an initially scattered configuration on an agarose surface. We track under the microscope the fates of hundreds of individual lineages, starting from individual ‘founder cells'. In our system, cells do not die and are not motile, so that the key processes are expansion competition and direct competition at patch boundaries. By defining a measure of competitive success based on the final area occupied by a lineage, we can identify those lineages that are ‘winners' and ‘losers', and determine the factors leading to their competitive success (or failure). We find that both expansion competition and boundary competition can play a role in colonization success, with their relative importance being dependent on the founder cell density. More specifically, a picture emerges from our experiments, supported by our simulations, in which the competitive outcome is controlled by both founder cell lag time and ‘squeezing’ between growing microcolonies at their boundaries. We also find that the emergent spatial patterns are density-dependent, with high initial cell densities typically leading to less uniformly shaped final patches. This study provides a baseline understanding of the factors that are at play when bacterial populations colonize soft surfaces, and a baseline methodology for investigating competition for space in microbial communities more generally.

## 2. Results

### 2.1. Visualizing the fate of individual cell lineages

**Figure 1.**
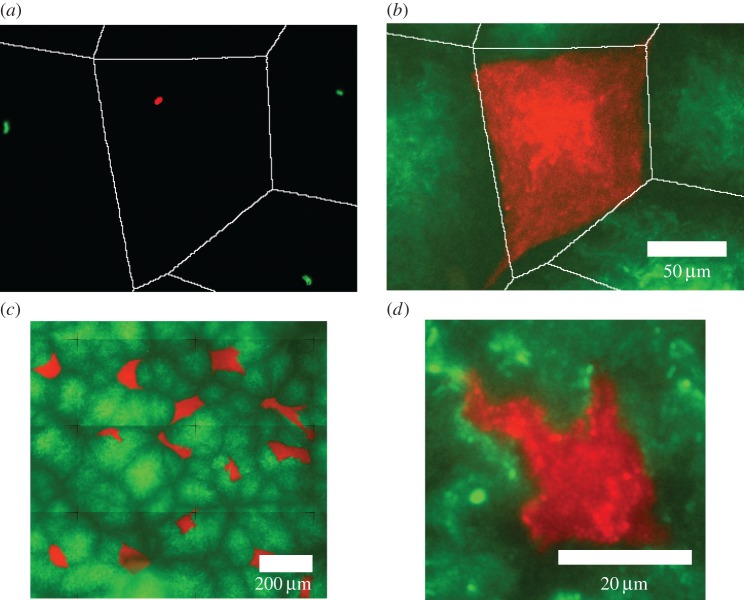
In our experiments, *E*. *coli* cells expressing two different fluorescent labels proliferate and compete for space. Cells expressing either cyan (CFP) or yellow fluorescent protein (YFP) are shown here as false colour red and green, respectively, to aid visual contrast. (*a*) An initial configuration of progenitor cells on the surface; Voronoi polygons (white lines) are generated based on the positions of the progenitor cells. (*b*) A patch that results from the proliferation of one of the individual cells shown in (*a*); the surrounding patches are green (YFP rather than CFP), so do not appear on this image. (*c*) A montage of several microscope fields of view, showing how a complete picture of the colonized surface can be built. The red patches in these types of image are analysed to produce the results presented in this paper. Variations in fluorescence intensity are caused by variation in cell density across the agarose surface. (*d*) A detail of a patch arising in an experiment with an initially higher cell density, which shows a less uniform patch shape. In all images, the brightness levels have been adjusted in ImageJ for clarity.

To track the fate of individual cell lineages during surface colonization, we mixed two differently coloured fluorescently labelled strains of *E. coli* MG1655 (initially growing exponentially in liquid culture, see Methods) in a 1 : 19 ratio. A small volume of the mixed culture was spread evenly on the surface of a flat agarose pad containing nutrients, and the sample was sealed with a glass coverslip. We used fluorescence microscopy to record the positions of over 1900 of these ‘founder cells' (figure 1*a*) across spatial regions up to ≈2 × 10^5^ µm^2^. We then allowed the cells to proliferate during overnight incubation, so that the entire spatial domain became covered, before re-imaging (figure 1*b–d*). Figure 1 shows typical results, with the minority and majority strains shown in red and green, respectively. After the surface is colonized, distinct patches of red cells can be seen, each of which is surrounded by a ‘lawn’ of green cells. Each red patch corresponds to space that has been occupied by the progeny of an individual red founder cell, in competition with the surrounding green cells. A 1 : 19 ratio was chosen to minimize the number of CFP colonies sharing collision boundaries. By analysing the sizes and shapes of the red patches, we can investigate the outcome of competition for space among the founder cells.

Figure 1*b*,*d* shows the examples of individual red patches, in experiments with low (figure 1*b*, *ρ*_low_ = 1.5 × 10^−4^ cell µm^−2^) and high (figure 1*c*, *ρ*_high_ = 7.2 × 10^−3^ cell µm^−2^) average densities of founder cells. For low founder cell density, we typically obtain patches with straight edges and clearly defined corners. For high founder cell density, patch shapes tend to be more ragged.

### 2.2. Quantifying winners and losers

To quantify the outcome of competition for space, we need to measure the extent to which a given cell lineage succeeds in outcompeting its neighbours. Starting from the initial configuration of founder cells, we assign a spatial ‘zone’ to each founder cell by performing a Voronoi tessellation based on the founder cell positions (see Methods). Each cell's zone consists of the region of space that is closer to it than to any other founder cell [38]. This partitioning is illustrated in figure 1*a*. As a null hypothesis, we suppose that, once the surface is colonized, the progeny of each founder cell will occupy the space defined by its zone. Comparing the shapes of the colonized patches which we observe in our experiments, we see that for low founder cell densities, there is indeed often a close correspondence between patch shape and the zones defined by the Voronoi tessellation (compare, for example, figure 1*a*,*b* which show the same region of space before and after colonization). For high founder cell density, colonized patch shapes tend to deviate more from the shapes of the Voronoi zones (e.g. Figure 1*d*). A more detailed, quantitative analysis confirms that shapes of the colonized patches and of the corresponding Voronoi zones correlate more strongly at low founder cell density than at high founder cell density (electronic supplementary material, figures S1 and S2).

To identify those cells that are ‘winners' and ‘losers' in the competition for space, we define a quantity that we call the ‘winner index’ (WI). This is the ratio between the colony patch area which arises from a given founder cell, and the area of its initial Voronoi zone2.1
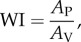
where *A*_P_ is the final patch area arising from a given founder cell, and *A*_V_ is the area of its Voronoi zone. A value WI = 1 corresponds to a cell whose lineage has colonized only the space closest to it. ‘Winners', or cells that colonize more space than that closest to them, will have WI > 1, whereas ‘losers', which colonize less space that that closest to them, will have WI < 1. Equation (2.1) does not, of course, provide the only possible definition of competitive success.

**Figure 2.**
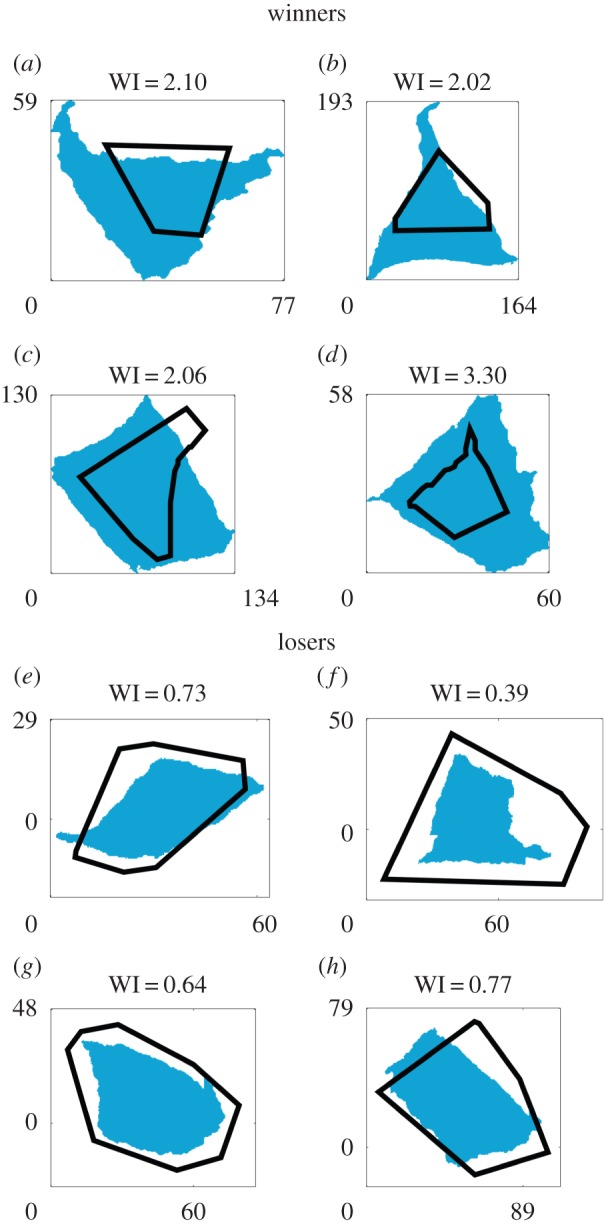
Shapes of selected patches resulting from ‘winner’ (*a–d*) and ‘loser’ (*e–h*) cells (blue), together with the shapes of their Voronoi zones (black lines) and their WI values. The blue shapes are obtained by thresholding the fluorescent images obtained after colonization. These results are taken from the low cell density experiment (*ρ*_low_ = 1.5 × 10^−4^ cell µm^−2^). Axes labels are micrometres.

Figure 2 illustrates the shapes of the colonization patches (blue) of several ‘winner’ and ‘loser’ cells, together with the shapes of their Voronoi zones (black lines), in an experiment with an initially low cell density (*ρ*_low_ = 1.5 × 10^−4^ cell µm^−2^). These images do not reveal any obvious ‘winning or losing strategy’; for example, while some winners seem to have gained territory by pushing out from their corners (figure 2*a,b*), others seem to have expanded more uniformly relative to their Voronoi zones (figure 2*g,h*).

**Figure 3.**
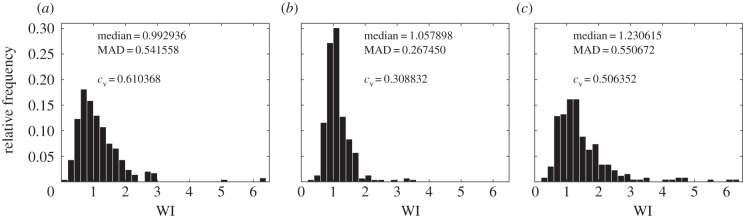
The distribution of winner index values across cells in the population is affected by our experimental conditions. WI distributions are shown for the following experiments, where *N* is the total number of matched colony–Voronoi pairs used in analysis. (*a*) High initial cell density (*ρ*_high_ = 7.2 × 10^−3^ cell µm^−2^), exponential growth, *N* = 274, taken from one mapped agarose pad. (*b*) Low initial cell density (*ρ*_low_ = 1.5 × 10^−4^ cell µm^−2^), exponential growth, *N* = 340, pooled from five mapped agarose pads. (*c*) Heat-shocked cells, low initial cell density (*ρ*_low_ = 1.5 × 10^−4^ cell µm^−2^), *N* = 313, pooled from three agarose pads. Two measures of distribution width are quoted in the panels: the median absolute deviation (MAD) and lognormal coefficient of variation (*c*_v_).

Figure 3*a*,*b* shows the distribution of WI values obtained in our experiments, for founder cells at high and low initial cell densities, respectively. In both cases, the distribution of WI values is peaked around WI = 1, but we also observe significant numbers of winners and losers. Comparing the results for low and high founder cell densities (figure 3*a*,*b*), we see that the distribution is broader at high founder cell density. For high founder cell density (*ρ*_high_ = 7.2 × 10^−3^ cell µm^−2^), the lognormal coefficient of variation (*c*_v_) was *c*_v_ = 0.61; in contrast, for the low founder cell density experiment, *c*_v_ = 0.31. Thus, when the founder cell density is high, such that expanding lineages collide earlier, we obtain more deviation from the Voronoi patch shape (electronic supplementary material, figure S2 and figure 1*d*), and also observe more extreme effects of competition for space (i.e. more winners and correspondingly more losers).

### 2.3. Expansion competition can play a significant role

Ecological theory suggests that two competitive processes are likely to be important in determining the outcome of our experiments: expansion competition, in which clonal lineages ‘race’ to occupy empty space, and direct competition at the boundaries between neighbouring patches [2,36,37].

#### 2.3.1. Tracking the early stages of surface colonization

**Figure 4.**
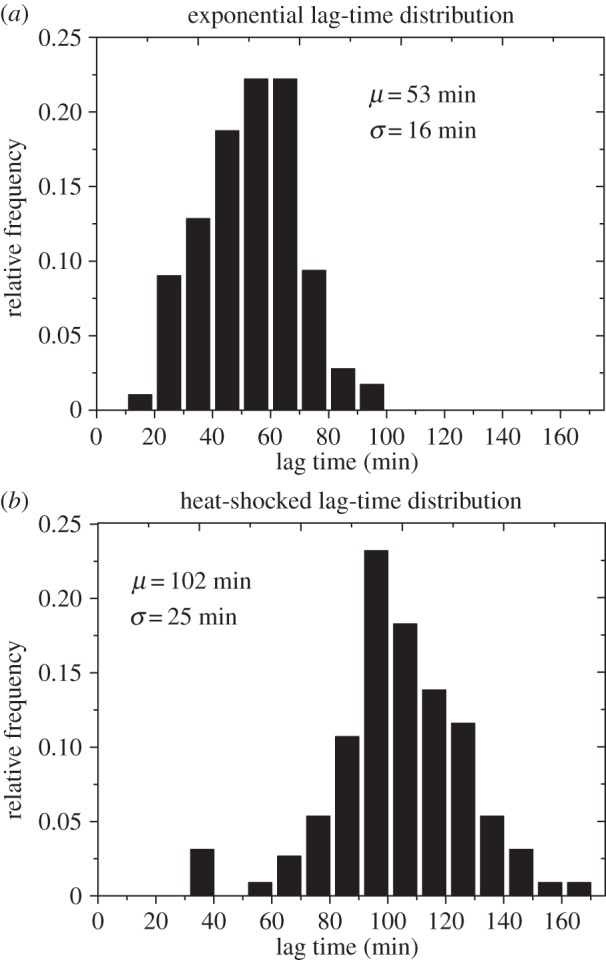
Founder cell lag times are broadly distributed and the width of the distribution can be manipulated by pre-treating the cells. Lag-time distributions are shown for (*a*) initially exponentially growing cells and (*b*) stationary-phase cells exposed to 50°C 15 min heat-shock prior to deposition on the surface. Mean lag times (*μ*) and their standard deviations (*σ*) are given in each panel. The heat-shocked cells show a longer average lag time and a broader distribution of lag times.

To determine the role of expansion competition in our experiments, we measured the growth behaviour of individual cell lineages (microcolonies) in the early stages of growth, i.e. prior to collision with their neighbours, using time-lapse microscopy. We measured the lag time, or waiting time before a founder cell starts to divide [39], and also tracked the rate of microcolony expansion, once a founder cell had started to proliferate. In agreement with others' observations [40–42], we observed significant variability in lag times among founder cells (figure 4*a*, mean *t*_lag_ = 53 min, with a standard deviation of 16 min). This variability is likely part owing to founder cells being at different stages of the cell cycle and intrinsic cell stochasticity (which is poorly understood) [43].

The expansion rates of growing microcolonies show much less variability: tracking the microcolony expansion rate as a function of microcolony size, we find that this varies somewhat during the first few divisions, but thereafter, the microcolonies expand exponentially with a mean area doubling time of 19.98 min and standard deviation of 0.12 min. In agreement with others [31], we also observe a transition between two growth regimes, each with a different expansion rate, at a microcolony size of about 5.3(8) × 10^3^ µm^2^; this has been shown to correspond to cells beginning to penetrate the agarose forming a second vertical layer (see electronic supplementary material, figure S4). For simplicity, our definition of WI does not take this vertical stacking into account; although it would be interesting to include it in future work.

#### 2.3.2. Manipulating lag times allows us to probe the effects of expansion competition

Because founder cell lag times are more variable than microcolony expansion rates in our experiments, we expect differences in the lag times of individual founder cells to be the dominant factor controlling expansion competition. To test the effect of lag-time variability, we manipulated the lag-time probability distribution. This can be achieved by using founder cells taken from the stationary phase of growth in liquid culture, which have been exposed to a sublethal heat shock (for 15 min), prior to deposition on the agarose surface (see Methods). This treatment has been shown in previous work to increase both the mean and variance of bacterial lag times [44–46], and this was, indeed, the case in our experiments (figure 4*a* compare with figure 4*b*). Sublethal heat shocks of Gram-negative bacteria are believed to disable important cellular components, with the biosynthesis of replacements causing a delay in cell growth [45].

#### 2.3.3. Broader lag-time distribution produces more winners and losers

If expansion competition is a significant factor in our experiments, we would expect that the broader lag-time distribution for heat-shocked founder cells should result in a broader distribution of WI values, with a higher proportion of lineages that either outcompete their neighbours (owing to relatively short lag times) or are outcompeted by their neighbours (owing to relatively long lag times). Thus, we expect our measure of WI distribution dispersion, *c*_v_, to be larger for the heat-shocked cells than for those deposited on the surface in the exponential phase of growth. This was, indeed, the case, as shown in figure 3: for heat-shocked founder cells, we observed that, for a founder cell density of 1.5 × 10^−4^ cell µm^−2^, *c*_v_ = 0.51, compared with *c*_v_ = 0.31 for the exponentially growing founder cells at the same cell density (compare the WI distributions in figure 3*b*,*c*). We assume that heat-shocking our cells only affects the cell lag times, because batch culture experiments showed population growth rates (*k*) in the exponential phase to be the same within error (e.g. at 32°C for RJA002, *k* = 0.0101(8) min^−1^ compared with *k* = 0.0110(4) min^−1^ for cells previously heat-shocked while in stationary phase. See the electronic supplementary material, figure S4).

### 2.4. Boundary competition is significant at high cell densities

Direct competition at the boundaries between colliding microcolonies may also be an important factor in our experiments. At these collision boundaries, local competitive interactions could, in principle, involve secretion of toxins or bacteriophage, extracellular polymeric substances or antibiotics [47]. For *E. coli* MG1655, however, cell–cell interactions within growing microcolonies on agarose surfaces are believed to be mainly mechanical—i.e. generated by physical pushing of the cells against each other, and by physical interactions between the cells and the agarose surface (as well as the glass coverslip) [25–31]. These mechanical interactions are expected to generate pushing forces between neighbouring patches as they collide, influencing the final colony shape.

#### 2.4.1. Correlating winner index with local neighbour geometry provides a probe of boundary competition effects

If boundary competition is a significant factor in our experiments, then we would expect the local geometry of the neighbours of a given founder cell to affect its eventual success. This local neighbour geometry is captured in the geometry of a founder cell's Voronoi patch—thus, by looking for correlations between Voronoi patch geometry and WI, we can investigate the influence of boundary competition in our experiments.

As a basic hypothesis, one might expect the *area* of the Voronoi patch to play an important role. On the one hand, cells with small patches need to gain less absolute area to achieve a large WI (because WI depends on the relative area). On the other hand, however, founder cells with large patches can produce large microcolonies, which might exert a greater pushing force upon collision. One might also expect the *shape* of a cell's Voronoi patch to be significant, because this reflects the number and geometrical arrangement of neighbouring founder cells, which should affect the force balance at the boundary between colliding microcolonies. We therefore looked for correlation between the eventual success of a given founder cell (as measured by its WI value) and both the size and shape of its Voronoi patch.

#### 2.4.2. Founder cells with small Voronoi patches tend to be winners

**Figure 5.**
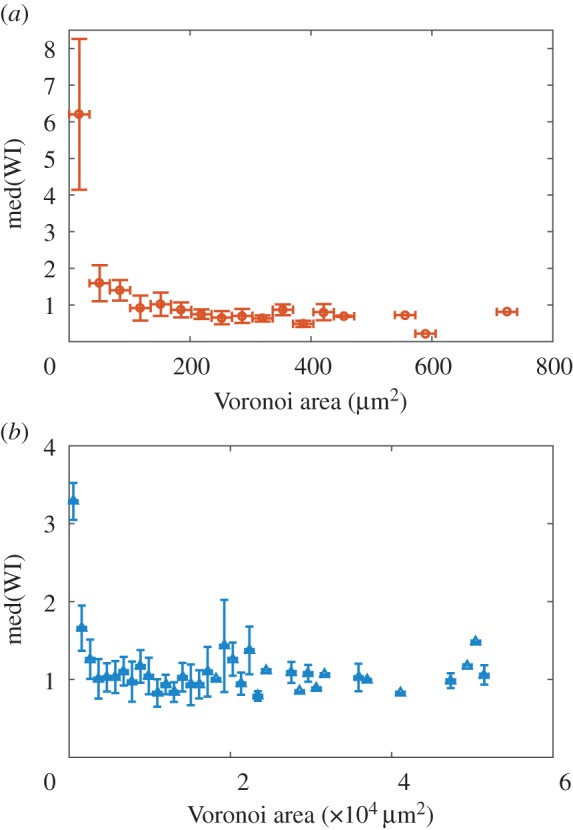
Cells with smaller initial patches are more likely to be winners. Median WI values are shown as a function of Voronoi patch size for (*a*) an experiment at high founder cell density (*ρ*_high_ = 7.2 × 10^−3^ cell µm^−2^), and (*b*) an experiment at low founder cell density (*ρ*_low_ = 1.5 × 10^−4^ cell µm^−2^). In both cases, founder cells were taken from exponential culture.

Our analysis showed that cells with smaller patches tended to have a higher average WI (figure 5). This was true for both the initial cell densities tested (in both cases for exponentially growing founder cells), although for the higher density (*ρ*_high_ = 7.2 × 10^−3^ cell µm^−2^; figure 5*a*), the trend was clearer. As discussed above, cells with smaller patches may tend to be winners simply because WI is a relative measure, so that cells with smaller patches need to gain less absolute area to be declared ‘winners'. However, a correlation between patch size and WI may also reflect the typical shapes of microcolonies when they collide. Because early-stage microcolonies are typically more anisotropic than late-stage microcolonies (see electronic supplementary material, figure S3), lineages with small Voronoi patches are likely to be more anisotropic when they collide with their neighbours. For example, at colony areas less than 1 × 10^3^ µm^2^, colony eccentricity (*e*) was on average *e* ∼ 0.8, whereas by 5 × 10^3^ µm^2^, *e* ∼ 0.35 on average. This might prove advantageous in ‘squeezing between the gaps' between colliding neighbouring microcolonies. Interestingly, we did not observe any advantage for founder cells with large Voronoi patches, even though these might be expected to generate a greater pushing force upon collision. We speculate that this is because larger microcolonies tend to have already ‘buckled’ into the vertical direction when they collide. In this case, newborn cells can be accommodated in layers, reducing the horizontal outward pressure within the microcolony [31].

#### 2.4.3. Voronoi patch shape differs between winners and losers

**Figure 6.**
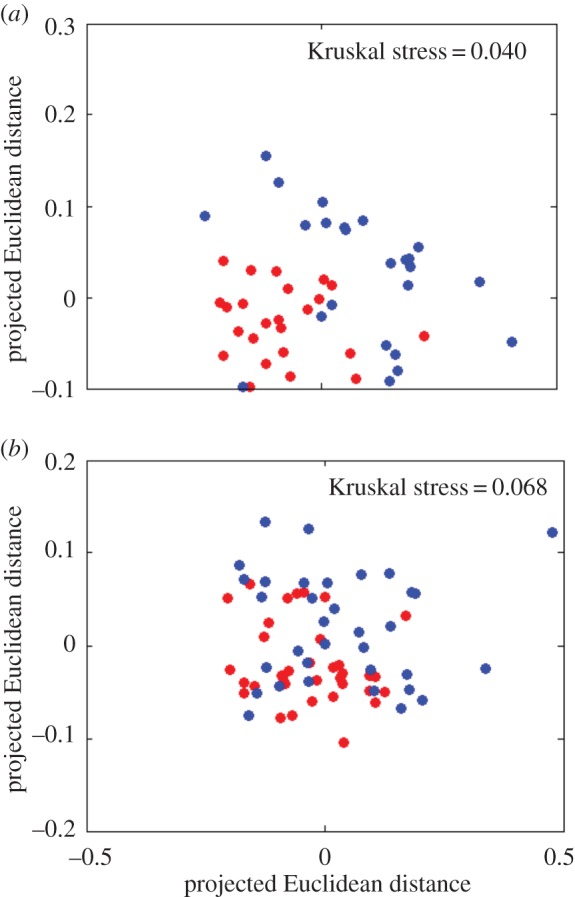
Founder cells that go on to be winners are statistically different in Voronoi patch shape from those that go on to become losers. MDS plots provide two-dimensional projections of the matrix of differences in Fourier descriptor spectra among individual Voronoi patches; the Kruskal stress measures the degree of agreement between the distances shown on the plots, and the actual computed distances. Red points represent cells that go on to be losers (bottom 10% of the WI distribution), and blue points those that go on to become winners (top 10% of the WI distribution). Distinct clusters of red and blue points indicate distinct typical Voronoi patch shapes for losers and winners. The statistical significance of this clustering was analysed by PERMANOVA (see Methods). (*a*) Results for high initial cell density, *N* = 48 (clustering significance by PERMANOVA: *p* = 0.001). (*b*) Results for low initial cell density, *N* = 70 (clustering significance by PERMANOVA: *p* = 0.008).

To explore the effects of Voronoi patch shape, we also characterized the shapes of individual patches, by computing their Fourier descriptor (FD) spectra [48]. The FD spectrum of a given shape is a list of numbers (FD magnitudes) that describe the amplitudes of different Fourier modes associated with the perimeter of the shape (for details, see Methods). The FD spectrum of an object depends only on its geometrical shape (it is independent of scaling, translation or rotation [48]). Thus, we can describe the shapes of the Voronoi patches corresponding to individual founder cells by their FD spectra. Importantly, this allows us to conveniently quantify the *difference* in shape between two Voronoi patches, by computing the Euclidean distance between their FD spectra (see Methods). We used this method to test for differences in the geometrical characteristics of winning versus losing Voronoi patches. Figure 6 shows our results, in the form of multidimensional scaling (MDS) plots for the high and low founder cell density experiments (figure 6*a*,*b*, respectively). An MDS plot projects the multidimensional matrix of shape differences between all pairs of patches onto two dimensions. In these plots, each point represents a founder cell Voronoi polygon and the distance between two points represents, as closely as possible, the dissimilarity between their shapes (as measured by the Euclidean distance between their FD spectra). Thus, founder cell points that are shown close together are similar in their Voronoi patch shapes while those that are far apart are different in their Voronoi patch shapes. In these plots, we have separated winners and losers by colour; the red points represent founder cells that eventually become ‘losers'—i.e. those whose eventual WI is in the lowest 10% of the population, and the blue points represent founder cells that go on to become ‘winners'—i.e. those whose eventual WI is in the highest 10% of the population. Clustering among either red or blue points in the plot would indicate a ‘typical’ Voronoi patch shape for either losers or winners.

Figure 6 shows that we do indeed observe clustering between Voronoi patch shapes for eventual winners and losers (distinct red and blue clusters of points), for both the high and low founder cell density experiments (figure 6*a* and *b*, respectively), although the clustering is more marked in the high-density case. The statistical significance of this apparent clustering can be measured using multivariate ANOVA [49] (PERMANOVA; see Methods); this showed a significant distinction between the winner and loser points for both the high-density experiment (*p* = 0.001) and the low-density experiment (*p* = 0.008). This supports our hypothesis that there are indeed geometrical features of the neighbour configuration that predispose founder cells towards success or failure in the competition for space, irrespective of patch size, even though these features are not immediately apparent on visual inspection of winner and loser Voronoi patches (figure 2).

### 2.5. Simulations reproduce our experimental results

Our experimental results suggest that the eventual success of a given cell lineage in outcompeting its neighbours depends on both the lag time before the founder cell begins to divide, and on the size and shape of the founder cell's Voronoi patch, defined by its neighbours. To verify this picture, and to further investigate the interplay between these two factors, we performed computer simulations of a simple model.

In our simulations, the spatial habitat is represented by a two-dimensional square lattice of 500 × 500 sites. We assume that each site can be occupied by a single bacterial cell. Upon reproduction, a cell retains its original site and also produces a progeny cell which occupies a previously empty site; thus, each founder cell gives rise to a microcolony which expands in space. The simulation algorithm is described in detail in Methods. Briefly, at the start of the simulation, randomly chosen sites are populated by founder cells. Each founder cell begins to reproduce after a waiting time which is chosen from one of our experimental lag-time distributions (figure 4). The age at which a cell reproduces is chosen from a uniform distribution with range 18–22 min (based on experiments tracking the area growth of colonies on 3% agarose at early times). Upon reproduction, the progeny cell is placed at random into any of the eight sites surrounding its parent (i.e. the Moore neighbourhood), if empty. In most of our simulations, if none of these sites is empty, the progeny cell is instead placed on the perimeter of the microcolony. This mimics the fact that in our experiments, birth of new cells deep within the microcolony pushes existing cells outwards, expanding the microcolony area. If the microcolony becomes completely blocked by neighbouring cells, i.e. there are no empty lattice sites at its perimeter, its growth ceases. To mimic a scenario where cell growth could be inhibited deep within the colony (e.g. by nutrient limitation), we also carried out some simulations in which progeny cells could only be placed in sites surrounding the parent. We term these two scenarios ‘exponential area growth’ and ‘perimeter growth’, respectively.

Our simulations allow us to investigate systematically the role of expansion and boundary competition. By adjusting the lag-time distribution, we can control the extent of expansion competition. By adjusting the founder cell density, we can mimic our experiments at low and high density and control the extent of boundary competition. Finally, by switching between the ‘exponential’ and ‘perimeter’ growth regimes, we can vary the strength of ‘pushing’ interactions between microcolonies once they have collided.

Because the microcolony area is observed to increase exponentially in our experiments (electronic supplementary material, figure S4), we begin with the exponential area growth regime, in which progeny cells are placed at colony boundaries if the Moore neighbourhood of their parent is full.

#### 2.5.1. Effects of expansion competition

**Figure 7.**
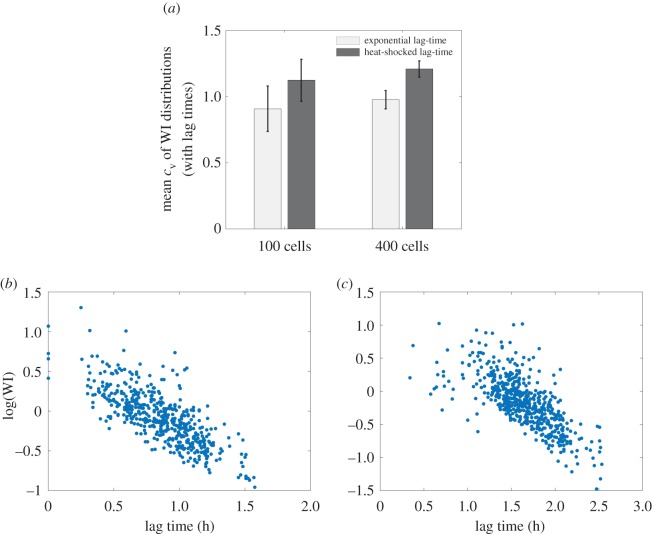
A broader lag-time distribution produces a broader winner index distribution, in our simulations as in our experiments. The lognormal coefficient of variation (*c*_v_) of the WI histograms is shown for simulations using different lag-time distributions and initial seed cell densities. Using the heat-shocked lag-time distribution (taken from our experimental data, figure 4*b*) results in a wider WI distribution than using the exponential lag-time distribution (figure 4*a*, two-way ANOVA *p* < 0.01). This result holds for two different founder cell densities, 100 initial cells (i.e. 4 × 10^−4^ cells per site) and 400 initial cells (i.e. 1.6 × 10^−3^ cells per site), although cell density did not affect mean *c*_v_ values for the same lag-time distribution (two-way ANOVA *p* = 0.12). In all cases, simulations are performed in the exponential area growth regime. (*b,c*) Log(WI) versus lag time for individual cells in our simulations, where the lag times assigned to each cell have been picked from an experimentally determined distribution. The lag times assigned in this case are ones measured in cells that had been picked from liquid culture while still in the exponential phase (*b*), or heat-shocked while in stationary phase (*c*) before being spread on an agarose surface.

We first tested whether our simulations could reproduce our experimental observation that a broader lag-time distribution led to a broader distribution of WI values (figure 3). To this end, we performed simulations (in the exponential area growth regime) with lag times sampled from our two experimental distributions: the lag-time distribution for exponential phase founder cells (figure 4*a*), and that for heat-shocked founder cells (figure 4*b*). As expected, we observed a wider distribution of WI values for the simulation with the broader (heat-shocked) lag-time distribution (figure 7). In our simulations, we are also able to correlate the lag times of individual founder cells with their eventual fate (i.e. their WI value)^1^. This analysis showed that indeed those cells with shorter lag times had, on average, higher values of the WI (figure 7*b,c* and electronic supplementary material, figures S5 and S6).

#### 2.5.2. Effects of boundary competition

**Figure 8.**
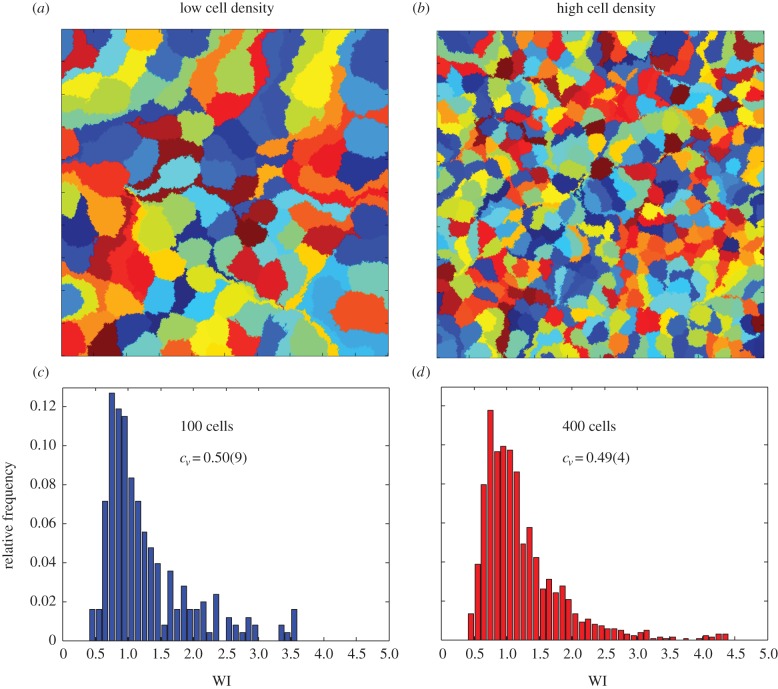
Snapshots of final simulation configurations, for simulations with no lag times, in the exponential area growth regime. In both panels, the lattice is 500 × 500 sites, with either (*a*) 100 seed cells or (*b*) 400 seed cells. Simulations without lag times produce a spread of winners and losers, suggesting that boundary competition is important. WI distributions are shown for simulations in the exponential area growth regime, without lag times, for two founder cell densities: (*c*) *N* = 100 seed cells, (*d*) *N* = 400 seed cells.

In our simulations, we can also eliminate the effects of expansion competition, by allowing all the founder cells to divide from the start of the simulation (i.e. removing the lag times, so that all colonies expand at equal exponential rates from the start). This allows us to investigate the effects of boundary competition in isolation. Figure 8 shows typical snapshots of the final configuration from such simulations (with different microcolonies coloured differently). In these simulations, final colony shapes are typically irregular, suggesting that there is an effect of ‘pushing’ at the boundaries between colliding colonies. This is also apparent in the WI distributions for these simulations (figure 8*c,d*), which are broad, showing that boundary competition has created significant numbers of winners and losers.

**Figure 9.**
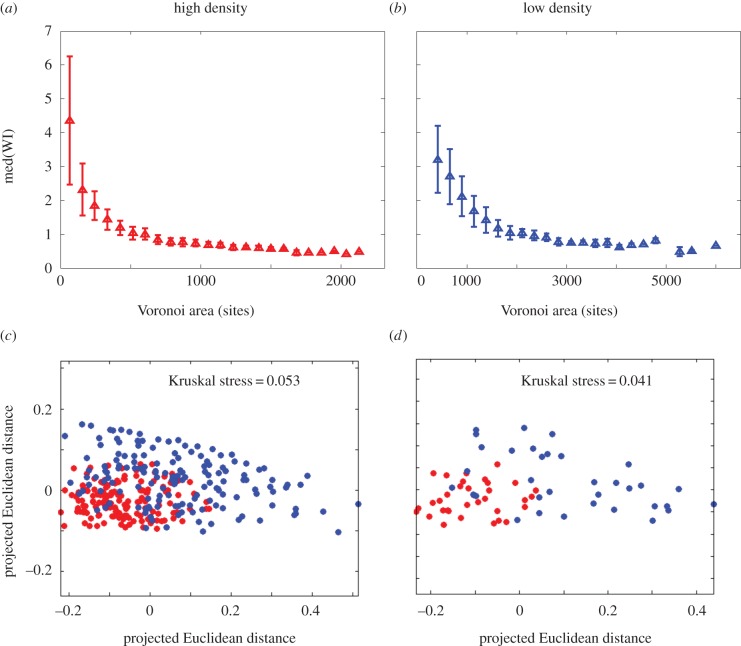
Simulations without lag times in the exponential area growth regime show similar effects of Voronoi polygon size and shape as in our experiments. (*a,b*) Median WI values are plotted as a function of binned Voronoi polygon area, for simulations with 400 (*a*) and 100 (*b*) founder cells. (*c,d*) MDS plots show the similarity between Fourier spectra of Voronoi polygons in the top (blue, ‘winners') and bottom (red, ‘losers') 10% of the relevant WI distributions, for simulations with 400 (*c*) (*N* = 308) and 100 (*d*) (*N* = 68) founder cells; the Kruskal stress measures the degree of agreement between the distances shown on the plots, and the actual computed distances. In both cases, clustering among the red and blue points is statistically significant (PERMANOVA: *p* = 0.001 for both low and high founder cell densities.

Repeating our analyses of Voronoi patch size and shape, we find that, as in our experiments (figures 5 and 6), in our simulations, founder cells with smaller Voronoi patches tended to be successful (figure 9), and there is clear clustering between the shapes of the Voronoi patches corresponding to ‘winning’ and ‘losing’ founder cells (figure 9, statistically significant clustering of winners and losers, *p* = 0.001 for both low- and high-density simulations).

Interestingly, however, founder cell density seems to have a slightly different effect in our simulations compared with our experiments. In the experiments, the distribution of WI values was broader for experiments at high founder cell density, compared with low founder cell density (figure 3*a,b*), and we also observed a more pronounced effect of Voronoi patch size and shape at high founder cell density, compared with low founder cell density (figures 5 and 6); although the effect of shape was statistically significant in both cases. In contrast, in the simulations, the distributions of WI values are similar in width for simulations at low and high founder cell density (4 × 10^−4^ and 1.6 × 10^−3^ cell site^−1^, or 100 and 400 initial seed cells, respectively), and we observe similar effects of Voronoi patch size and shape for the two founder cell densities (figure 9, left and right panels). One possible explanation for this might be that in the experiments, colonies that collide when they are small (as in the case of high founder density) are still growing strictly as a single layer in the horizontal plane, whereas colonies that collide when they are larger (as in the case of low founder cell density) are already growing as multiple layers [31], so exert reduced pushing forces in the horizontal plane. This factor is not taken into account in the simulations, but could be tested in future work.

### 2.6. ‘Pushing’ interactions between microcolonies play an important role in boundary competition

In our simulations, we can change the way that microcolonies interact at collision boundaries, by switching between the ‘exponential area growth’ and ‘perimeter growth’ regimes. In the former regime, microcolonies that collide will continue to grow exponentially, adjusting their shape to fit between the gaps until each microcolony is completely surrounded. In the latter regime, growth is locally inhibited at collision boundaries such that colliding microcolonies slow, and eventually stop, their growth. This regime might correspond to a case where nutrient penetration to the inner parts of the colony is a limiting factor [30], or might loosely mimic a case where colonies grow in several stacked layers [31].

**Figure 10.**
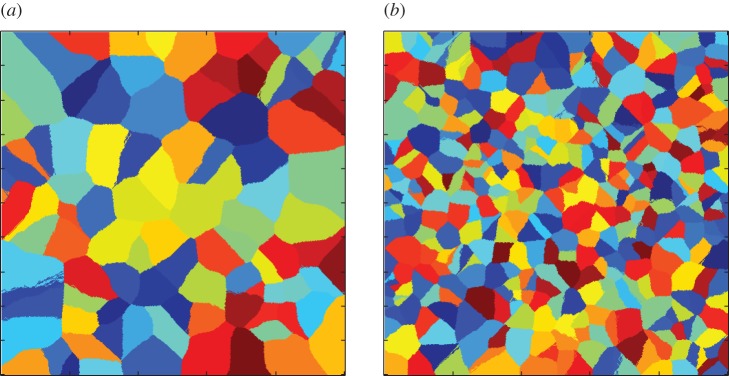
Snapshots of final simulation configurations, for simulations with no lag times, in the perimeter growth regime. In both panels, the lattice is 500 × 500 sites, with either (*a*) 100 seed cells or (*b*) 400 seed cells.

**Figure 11.**
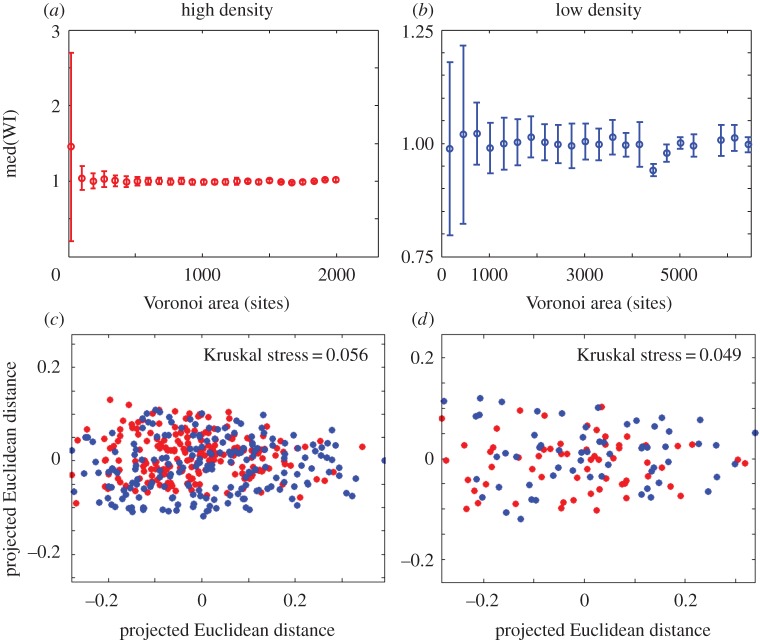
Simulations without lag times in the perimeter growth regime do not show significant effects of patch area or shape on the WI. (*a,b*) Median WI values are plotted as a function of binned Voronoi polygon area, for simulations with 400 (*a*) and 100 (*b*) founder cells. (*c,d*) MDS plots show the similarity between Fourier spectra of Voronoi polygons in the top (blue, ‘winners') and bottom (red, ‘losers') 10% of the relevant WI distributions, for simulations with 400 (*c*) (*N* = 394) and 100 (*d*) (*N* = 120) founder cells; the Kruskal stress measures the degree of agreement between the distances shown on the plots, and the actual computed distances. In both cases, clustering among the red and blue points is not statistically significant (PERMANOVA: *p* = 0.135 for high founder cell density and *p* = 0.202 for low founder cell density).

Repeating our simulations in the perimeter growth regime (again in the absence of lag times), we obtain quite different results. Figure 10 shows typical final snapshots from our simulations, for the same parameters as in figure 8, but in the perimeter growth rather than the exponential growth regime. Comparing figure 10 with figure 8, we see that in the perimeter growth regime, the final patch shapes are more regular, corresponding more closely to the Voronoi polygon areas associated with the founder cells. Analysing the effects of Voronoi patch size and shape on the final outcome of competition in this regime (figure 11), we also find that neither patch size nor shape are significant factors in distinguishing winners from losers (compare figure 11 with figure 9). These results suggest that microcolony ‘squeezing’ at the boundaries of colliding colonies may be responsible for the boundary competition effects (e.g. effects of Voronoi polygon size and shape) that we see in our experiments.

## 3. Discussion

In this paper, we have used a bacterial model system to investigate the ecological factors influencing competition for space as an initially scattered population colonizes a spatial domain. Our study has focused on a ‘simplest possible’ model system, in which the competing populations are identical (except for their fluorescent markers) and death, dispersal and complex ecological interactions are all absent. For this system, we can track the fates of individual founder cells as they proliferate, allowing us to define a simple measure of the outcome of competition, which we term the ‘WI’. Quantifying success in this way allows us to investigate in detail the factors that influence the outcome of competition for space, at the level of individual founder cells.

In our experimental system, competition for space involves expansion competition, manifested as differences in the founder cell lag times, and boundary competition, manifested as ‘pushing’ interactions between growing microcolonies as they collide at their boundaries. The former is detectable in an effect of changing the lag-time distribution (which we achieve by heat-shocking the founder cells), and the latter is detectable in the form of correlations between the founder cell's neighbour geometry and its eventual success. We find evidence for the importance of both these competitive mechanisms, but boundary competition shows more pronounced effects when the experiment is initiated with a high founder cell density, such that colonies collide when they are smaller. We speculate that this may be because small colonies are typically expanding asymmetrically and exponentially, in a single horizontal plane, whereas larger colonies are typically more symmetric in shape, may be nutrient-limited in their centres (although this is unlikely in our experiments) and are likely to consist of several layers of cells.

Importantly, we are able to reproduce the key features of our experiments using computer simulations of a simple model that includes only patch expansion (with a lag time) and exclusion between microcolonies at their boundaries. In our simulations, we can manipulate the growth rules to favour either expansion or boundary competition. Eliminating ‘squeezing’ of microcolonies as they collide with their neighbours, by restricting cell proliferation to the perimeter of expanding microcolonies, drastically decreases the effects of boundary competition in our simulations.

Our study is intended as a starting point in investigating competition for space among expanding microbial populations, and as such, naturally involves technical aspects that warrant further discussion. First, our analysis inevitably forces us to make a somewhat arbitrary definition of the WI. Second, we chose to end our analysis soon after the whole surface was colonized (i.e. after ≈16 h); one might wonder whether our results might change over a longer timescale, as cells continue to interact at microcolony boundaries. This would certainly be interesting but from a practical point of view, very long timescale experiments are difficult owing to the tendency of the agarose pad to dry out. Third, it is important to note that for larger microcolonies, a second layer of cells forms prior to collision with the neighbours; i.e. growth is occurring to some extent in the vertical as well as horizontal direction [31]. This is not taken into account in our definition of the WI. One could perhaps include it by measuring total fluorescence rather than patch area, or by performing confocal microscopy, but both involve their own technical challenges.

Despite these challenges, our results do provide a clear picture of the ecological factors influencing competition for space in this system. One of the interesting results that emerge from our study is that at low founder cell density, the patches colonized by individual lineages are in the main remarkably similar to the Voronoi patches corresponding to the founder cell neighbour configurations (figure 1). This is by no means an obvious outcome, because growing *E. coli* microcolonies are essentially granular materials, with complex internal cell orientations [25–31], which should lead to complicated force balances upon collision with neighbouring microcolonies. Indeed, some other studies of similar systems have reported ‘fractal-like’ patterns, in which colliding lineages form elongated, intertwined domains [21,32]. Although we do see greater deviation between final and Voronoi patch shapes at higher founder cell densities (figure 1), we do not observe fractal-like patterns, even at very high founder cell densities. Although this requires further investigation in future work, our set-up does differ from previous studies [21,32] in that the growing populations are confined by a glass coverslip that is firmly pressed down on them. If this does turn out to be important in determining the patterns formed, this would be an interesting manifestation of the importance of physical interactions (such as elasticity and friction) in determining the outcome of bacterial community organization [30,31].

To what extent can the approach described here be used to understand spatial competition in more complex ecological communities? Many of the complex interactions observed in macro-ecosystems exist in the microbial world; microbes show a plethora of dispersal strategies [50,51], life-history trade-offs involving lag times, survival probabilities, growth rates and yields [52–54], as well as social interactions including chemical signalling, quorum sensing, toxin and viral warfare [12,55]. The effects of these factors on the development of spatially structured communities could easily be investigated by varying the microbial species and strains that compete in our experimental set-up. Interestingly, we note that recent high-throughput macroscopic studies of growing colonies of bacteria extracted from soil arrived at a similar conclusion to ours concerning the importance of variation in founder cell lag times for eventual competitive success [40]; although for soil bacteria the situation appears to be more complex, because lag times were dependent on initial cell density [40]. More generally, natural microbial communities often show extremely high levels of species diversity, and as yet, we lack basic fundamental understanding of how this diversity arises and is maintained, and what functional role it may play. Well-controlled laboratory experiments, in conjunction with ecological theory, are essential if we are to change this state of affairs [13].

## 4. Experimental methods

### 4.1. Bacterial strains and growth conditions

Two strains of *E. coli* MG1655 were used in our experiments, one carrying a cyan fluorescent protein (CFP) reporter construct and the other a yellow fluorescent protein (YFP) reporter construct. These strains were made by P1 transduction from strain MRR of Elowitz *et al.* [56] into an MG1655 background. The reporter constructs are integrated into the *E. coli* genome at positions on either side of the origin of replication (equidistant from the origin) and are under the control of the constitutive *λ*P_R_ promoter [56]. Separate overnight cultures for each strain were prepared by transfer of a single isogenic plate colony (themselves sourced from freezer stocks (LB + 30% glycerol)) into 5 ml M9 medium, supplemented with glucose (0.4%) and casamino acids (0.5%; all further references to M9 include these supplements) and incubated for approximately 16 h at 37°C, 200 r.p.m., where they reached the stationary phase. Growth curves obtained from plate reader data showed no significant difference in growth rates between the two strains.

For experiments using founder cells in the exponential growth phase, a 10^−3^ dilution was performed on each strain overnight into fresh M9 medium, and cells were grown to an optical density 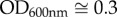. Heat-shocked cells were prepared by pipetting 1.5 ml of stationary phase overnight culture into an Eppendorf, and placing it in a 50°C heat-block for 15 min. Liquid cultures were mixed at a 1 : 19 (CFP : YFP) ratio, gently vortexed and diluted to the target optical density (which determined the resulting founder cell density on the surface) using fresh M9 medium.

In a typical experiment, we obtain between 20 and 100 red patches per agarose slab (see ‘sample preparation’ below), depending on the initial cell density. Any cases where patches arising from two separate red founder cells share an interface were discarded in our analysis.

### 4.2. Sample preparation

A sterile 55 × 25 × 2.4 mm Perspex slide with a 33 mm × 10 mm hole was lightly fixed to a standard glass microscope slide using petroleum jelly, creating a 792 µl well. Molten 3% M9 agarose was pipetted into the cavity and a microscope coverslip used to flatten the surface. Once the agarose had solidified, the coverslip was carefully removed, revealing an agarose slab. One microlitre of diluted mixed culture was pipetted three quarters up the length of the agarose, and the slide held at an angle, so liquid culture would run the length of the surface.

After the liquid culture had been fully absorbed, a clean coverslip was carefully placed over the agarose and sealed to the Perspex frame using VALAP [57]; the Perspex frame was then also sealed to the microscope slide to ensure air tightness.

For cell lag-time experiments, the Perspex slide was replaced with a Gene Frame (Thermo Scientific), a disposable, double-sided adhesive plastic frame with volume of 125 µl [58]. The cell mixing ratio was 1 : 1, and cultures were diluted to an appropriate optical density to maximize the number of cells in a single field of view.

### 4.3. Microscopy analysis of dual-fluorescence competition experiments

In our experiments involving mixtures of CFP and YFP-expressing cells, image acquisition was performed using fluorescent microscopy with filters appropriate for detection of either CFP or YFP. An inverted Nikon Ti Eclipse epifluorescent microscope, with 20× objective (NA = 0.75 or 0.5) and CoolSNAP HG^2^ (Photometrics) CCD camera at 2 × 2 binning were used for all samples. Excitation light was from a mercury lamp, passed through Chroma EYFP and ECFP filter sets. Automatic shutters controlled by MetaMorph (Molecular Devices) limited sample exposure to excitation light. A motorized stage (Prior Scientific) facilitated a precise raster scan of the sample, so relative positions of seed cells were accurately known. Following identification of seed cell positions (‘day 1’), the sample was placed in an airtight container and statically incubated overnight at 30°C, before repeating the imaging process (‘day 2’) to record the resulting colonies.

### 4.4. Time-lapse microscopy

Time-lapse microscopy was used to study the growth of microcolonies prior to collision; here, only a single strain was used. All samples were incubated at 32°C during image acquisition. Fluorescence and phase contrast time-lapse microscopy was performed on an upright Nikon E800 epifluorescent microscope with a 100× objective (Nikon, NA = 1.3, Ph3), and CCD camera (QImaging Retiga 2000R) at 1 × 1 binning. Excitation light was provided by a mercury lamp passed through appropriate filter sets. The microscope's XYZ-stage and shutters were controlled via µManager [59], and image acquisition automated using the inbuilt ‘OughtaFocus' autofocus algorithm. Multiple fields of view were observed in time by saving numerous XY-stage coordinates in the software.

### 4.5. Image analysis

Images were thresholded in ImageJ [60], and multiple fields of view were stitched together to form a complete, high-resolution montage of the entire surface from both day 1 and 2. The Voronoi map was generated in MATLAB using the built-in ‘Voronoi’ plugin, before being overlaid with a montage image of CFP cells, so Voronoi polygons generated by CFP cells could be identified. The CFP colonies and Voronoi polygons were then paired by identifying overlapping objects in the two images, using routines written in MATLAB^®^ (2012a, The MathWorks).

For cell lag times, phase contrast time-lapse movies of dividing cells were manually checked for division times using CellCounter in ImageJ, and corresponding fluorescent images used to determine if the cell expressed CFP or YFP.

### 4.6. Computation of Fourier descriptors

The complex FD method provides a convenient way to characterize Voronoi patch shapes. FDs are commonly used in shape recognition analysis [48,61]. A complete description of the boundary of the shape in frequency space is captured by taking the Fourier transform of the two-dimensional boundary coordinates, expressed as a real-complex number pair. This analysis results in a list of FD magnitudes corresponding to a given shape. Here, higher frequency terms (higher-order FDs) describe smaller features of the shape, whereas lower frequency terms describe larger, more coarse-grained features. The FD spectrum of a shape is translation, rotation and scale invariant.

To compute the FD spectra of our Voronoi patches, patch-boundary pixel coordinates were extracted in MATLAB and ordered so they formed a closed loop of *M* pixels. Vectors were created to store the coordinates *x_m_* and *y_m_* of each boundary pixel (where *m* denotes the index in the vector of boundary pixels for a given patch). Because an even number of boundary points is needed in the discrete fast Fourier transform algorithm [48], the last boundary coordinate was duplicated if necessary.

The real and imaginary parts of the FDs, *a_n_* and *b_n_*, were calculated from the boundary coordinates using4.1
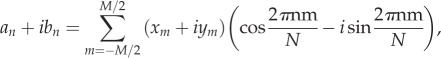
where *n* is the descriptor number and *N* the total number of descriptors [61]. We then obtained the FD magnitude *f_n_* for each index *n*, using 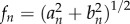. Descriptors of order 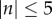 were used in analysis [61]. The list of *f_n_* values for each Voronoi patch was used to characterize its shape.

### 4.7. Quantifying differences in Voronoi patch shape using Fourier descriptors

To determine if there was a difference between Fourier signatures for each Voronoi polygon associated with a loser or winner progenitor cell, MDS analysis was used. Pairwise Euclidean distances between each Fourier spectra were used to build a dissimilarity matrix, quantifying the difference in shape between pairs of Voronoi polygons associated with a given experiment. Note that in this analysis we included only patches that fell into either the top or bottom 10% of the WI distribution (i.e. ‘winners' or ‘losers'). A two-dimensional projection of the dissimilarity matrix was created in MATLAB, and the points colour coded by their status as either winners or losers. An MDS plot was then generated from this two-dimensional projection, in which each point represents an individual Voronoi polygon and the distance between any pair of points represents the difference in their shape. To analyse the statistical significance of the apparent clustering visible in these plots (figure 6), we used PERMANOVA, implemented in the software Primer [49]. PERMANOVA analysis involves making multiple permutations of the dataset, randomly assigning colour labels. For each permutation, the average distance between points *within* and *between* colour sets is computed, and a distribution is computed for the ratio of these quantities. Comparing the ratio computed for the actual (non-permuted) dataset with this distribution allows one to assign a statistical significance to the apparent clustering seen in the MDS plot.

### 4.8. Computer simulation methods

Our discrete event computer simulations used a two-dimensional grid of 500 × 500 spatial lattice points, as described in the main text. We assumed that an individual bacterium occupied one lattice site, and bacteria were not allowed to overlap.

First, seed-cell coordinates were generated via a random number generator. From this, a list of cells was created (henceforth referred to as the ‘cell list’), each with an index number (which later was used to index the cell lineage). In our simulations with lag times, these were sampled from the experimental lag-time distributions and assigned to seed cells at random; the cell-list was then sorted in ascending order by these lag times. If no lag time was used, then division times were assigned instead, picked from a uniform distribution with range 18–22 min, and the cell list sorted by these division times. This range was determined from area growth rates derived from time-lapse movies of isolated microcolonies (see electronic supplementary material, figure S4).

Simulation updates were performed by jumping directly to the next division event in time. In practice, this meant looping over the cell list, previously sorted in chronological order, where each iteration of the loop encapsulated the division of one cell, with the top-most cell in the cell list the current mother cell undergoing division. Each ‘timestep’, therefore, was the difference in division time between the current and previous cell to divide, and the system state was assumed to remain unchanged between each cell division event.

For each simulation update, the top-most cell in the cell list would be dividing (the mother cell). To decide where a daughter cell would be placed, any unoccupied eight-way connected lattice sites were first checked, and one chosen at random for occupation. Both the daughter and mother cell were then assigned new division times, and sorted back into the main cell list, and the next iteration of the loop advanced, where a new mother cell would undergo division. However, if the mother cell had no free adjacent lattice sites available, the daughter cell was instead placed on the edge of the colony. To do this, a list of empty lattice sites on the edge of each colony was maintained. From this list, empty lattice sites which were within a threshold distance (defined by the user) were selected. Changing the threshold value allowed control of the manner of colony growth, such as perimeter only growth, or exponential area growth. Both daughter and mother cells were then assigned a new divisions time and sorted in the main cell list. If no available sites existed, then the mother cell was permanently removed from the cell list. If a mother cell tried to place a daughter cell beyond the boundary edge, it was allowed, but the new cell was not tracked. This way, the edges were treated as open, and did not confine growth. This is similar to the experimental set-up, where a microscope has a finite field of view which colonies at the edges can grow beyond. However, because we did not then know the true area of colonies that touched the edge of our simulation ‘field of view’, these colonies were not used in analysis. The simulation finished once all lattice sites were occupied by cells.

## Supplementary Material

Supplementary Information

## Endnote

1 Correlating lag times of individual cells with winner index values is not possible in our experiments, due to insufficient objective resolution.

## Data accessibility

Datasets are available for download through the following link at http://goo.gl/dQlqNV.
