# Supplementary material for: Competition for space during bacterial colonization of a surface
Source: J R Soc Interface. 2015 Sep 6;12(110):20150608. doi: 10.1098/rsif.2015.0608 (PMC4614474; doi:10.1098/rsif.2015.0608)
Supplement: Supplementary Information [file rsif20150608supp1.pdf]

Supplementary Material: Competition for space during bacterial colonisation of a surface

Diarmuid P. Lloyd and Rosalind J. Allen
SUPA, School of Physics and Astronomy, University of Edinburgh, James Clerk Maxwell Building, Peter Guthrie Tait Road, EH9 3FD, Edinburgh, United Kingdom

I. VORONOI AREA DISTRIBUTIONS

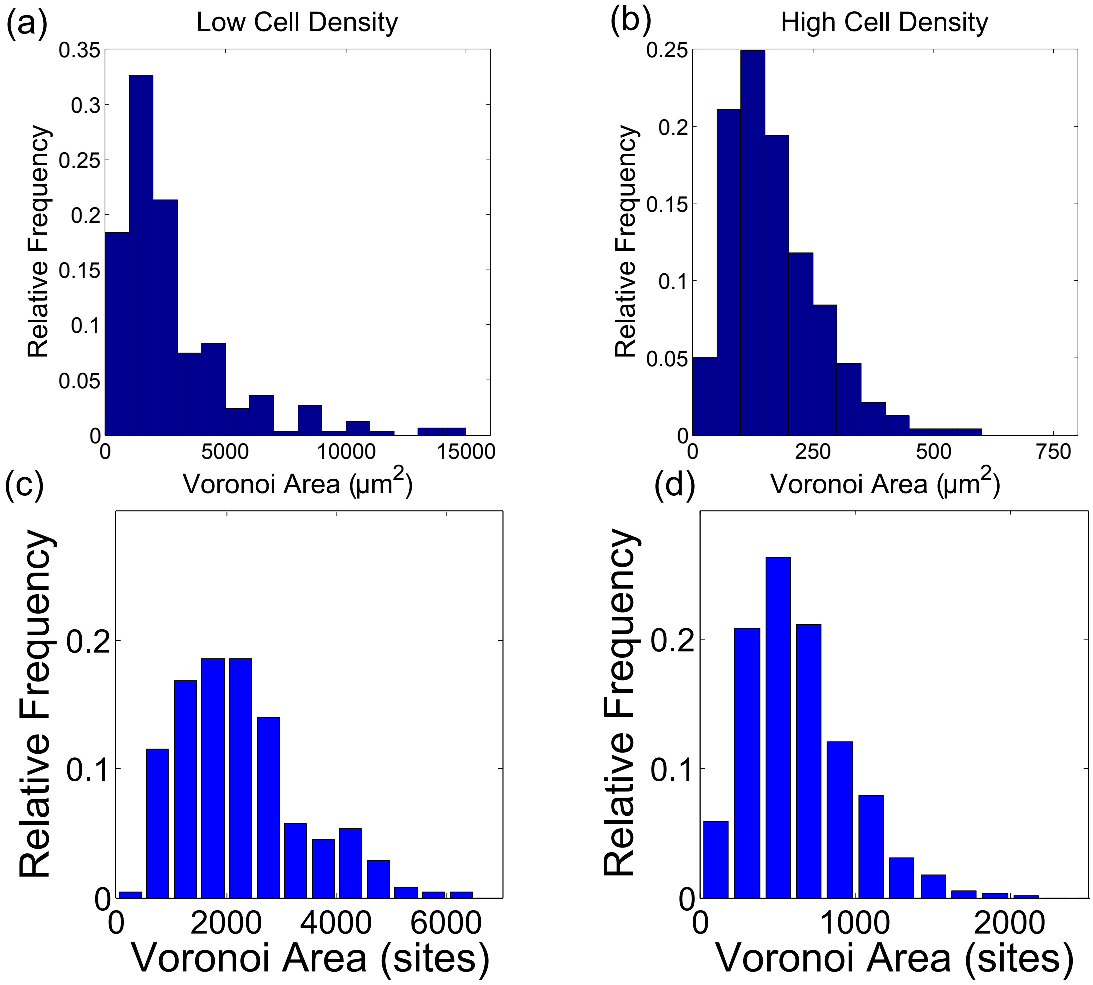

Figure 1: Voronoi area distributions in the (a) 'low' (ρ_{low} =1.5 × 10^{-4} cell/μm^2) and (b) 'high' (ρ_{high} =7.2 × 10^{-3} cell/μm^2) density experiments, and (c) 'low' (4 × 10^{-4} cell/site) and (d) 'high' (1.6 × 10^{-3} cell/site) density simulation runs.

Adjusting the cell density when seeding the agarose surface allowed us to adjust the distribution of Voronoi polygon areas. Fig1(a,b) shows the distributions of Voronoi areas for the "low" and "high" density experiments, showing an almost order of magnitude difference in the average areas obtained for Voronoi patches in the two experiments. Similarly, Fig1(c,d) shows Voronoi area distributions for the typical cell densities tested in the simulations. The optical densities of liquid culture used to seed the agarose pads (providing the Voronoi area distributions) in our experiments are summarised in Table I, and Table II contains the initial cell density across the 500x500 lattice in the simulations.

Experiment	OD ranges
Low density	0.005, 0.008, 0.01, 0.015
High density	0.554

Table I: Optical density (600 nm) measurements for the liquid cultures used to inoculate the agarose slabs in our low and high density experiments.

Simulation type	Seed cell number	Cell density (cell / total sites)
Low density	100	0.0004
High density	400	0.0016

Table II: Initial cell densities used in our simulations.

II. VORONOI POLYGON AND COLONY SHAPE CORRELATIONS

Qualitatively, we noticed that larger colonies tended to match their Voronoi polygons more closely compared to smaller ones. These effects can be seen by computing the degree of correlation between FD magnitudes for colony-Voronoi pairs. A high degree of correlation implies strong similarity in shape between the Voronoi patch and the final colony. Fig. 2 shows the correlation coefficients for the high and low density experiments, for each Fourier descriptor. Generally, colony-Voronoi polygon pairs in the lower density experiment matched in their shape descriptors more than in the higher density cases.

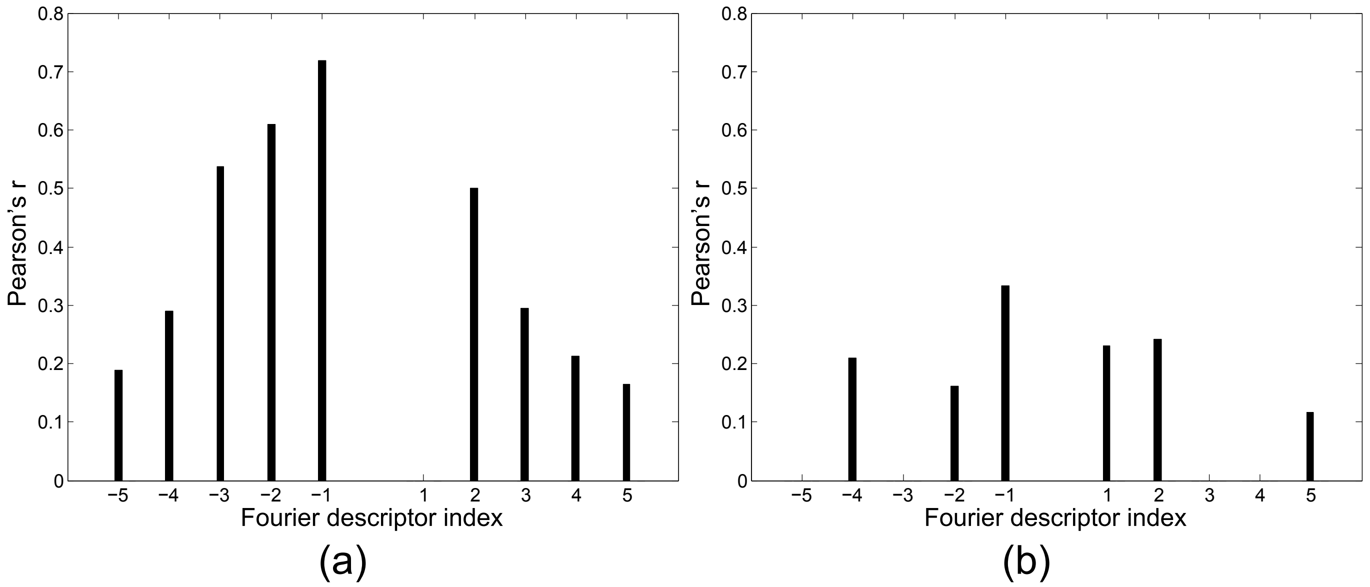

Figure 2: Pearson correlation coefficients measuring the extent to which Fourier descriptor magnitudes describing the shapes of Voronoi polygons and final colony shapes match each other in the (a) low and (b) high cell density experiments. Only coefficients at the $p < 0.1$ significance level are shown.

III. COLONY ANISOTROPY AND GROWTH RATES

Growing on 3 % agarose, colonies arising from individual founder cells initially underwent anisotropic expansion, but eventually approached a circular shape as they grew larger (Fig. 3). Colonies have similar area expansion growth rates (inital area doubling times measured at 19.98(12) min). In agreement with previous work[1] we observe a transition in the area growth rate at a well-defined colony area (Fig. 4). This is believed to correspond to invasion of the agarose in the vertical direction to form a second cell layer.

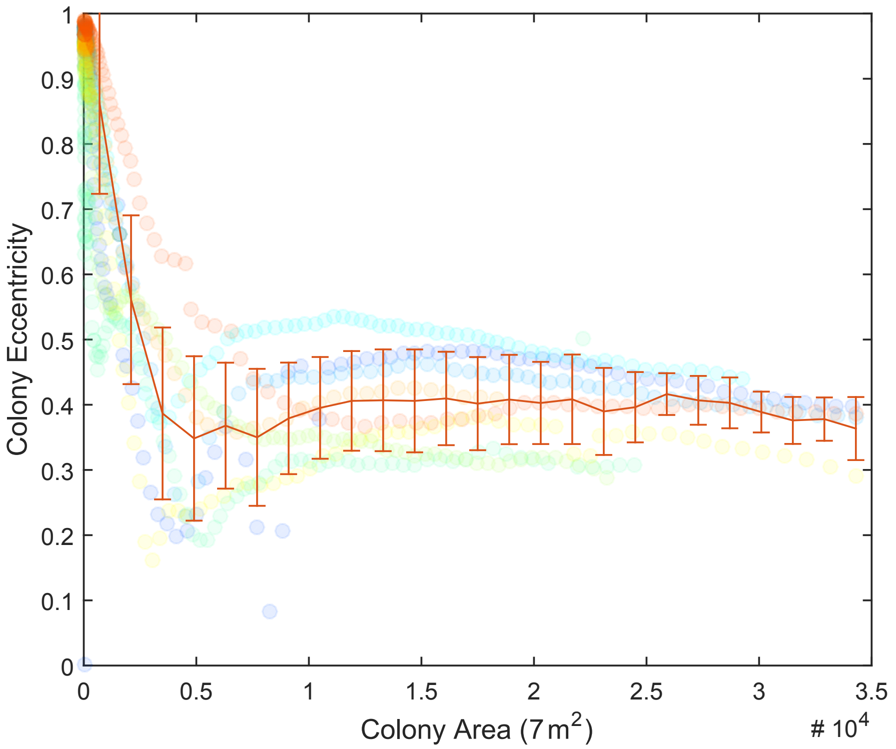

Figure 3: Colony eccentricity (e) measurements for 7 different colonies growing on 3 % agarose (filled circles), and an average curve, calculated from binning (bin width, 1400 µm^2) the individual colony areas and taking the mean e. Error bars are the standard deviation. The eccentricity, measured using MATLAB's `regionprops` function, is the aspect ratio of an ellipse, with the same second moments as the colony shape. A value of 1 corresponds to a line segment, and 0 a circle.

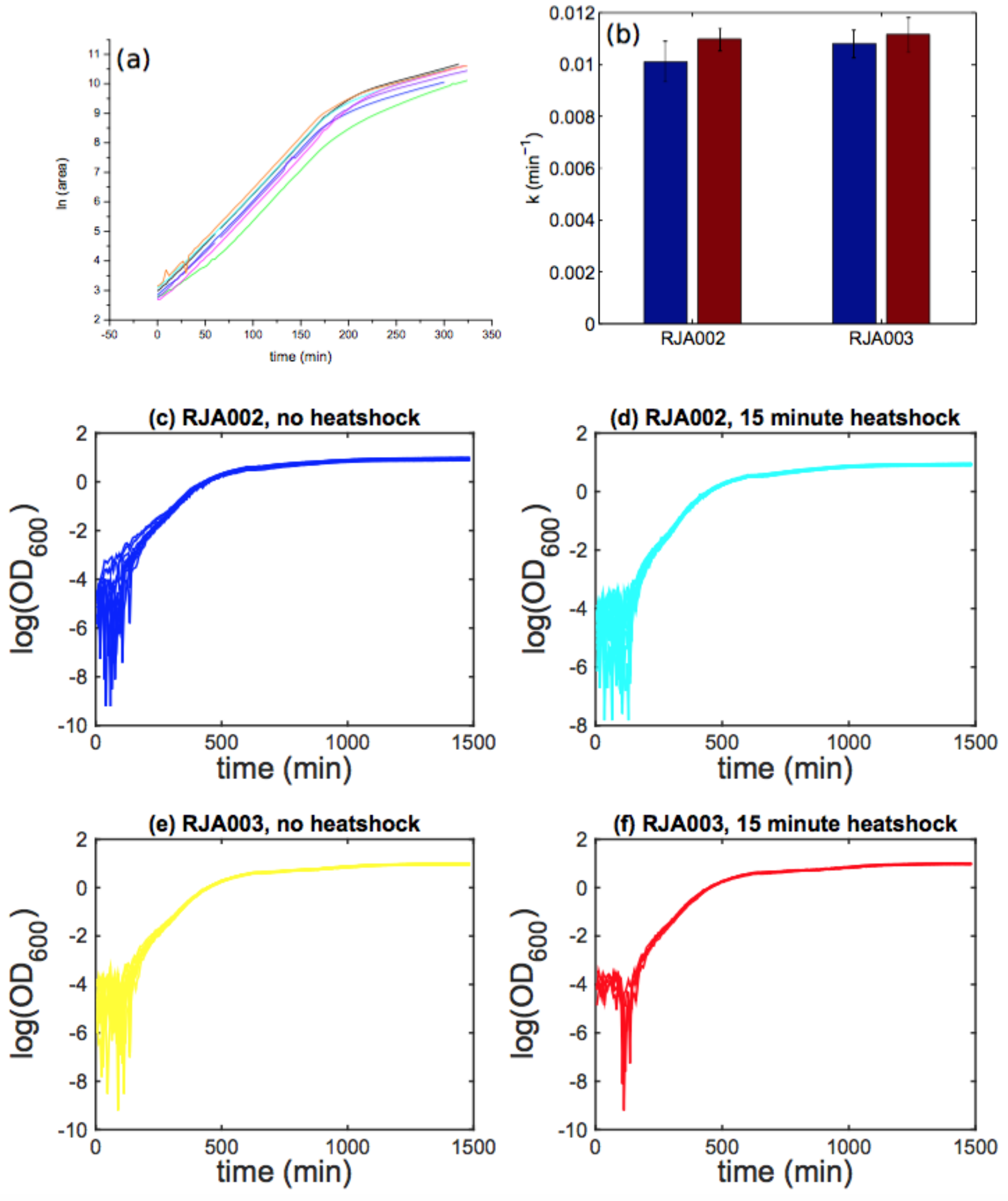

Figure 4: (a) Colony area growth curves for 8 different colonies growing on 3 % agarose. From least-square linear fits to the pre-invasion regions of the curves ($t \leq 150$ min), a mean colony area doubling time of 19.98(12) min at 37 °C was obtained. (b) Mean growth rates obtained from OD measurements of batch culture of the two strains at 32 °C, for either no heat-shock, or heat-shock while in stationary phase treatments. Heat-shocking resulted in no significant difference in the mean growth rate while in the exponential phase (c-f). Growth curves used to calculate the exponential growth rates presented in panel b. Linear fits in the least square fits were made to regions of the curves where it was deemed representative of the exponential growth phase.

IV. CORRELATION BETWEEN INDIVIDUAL LAG TIMES AND WI IN OUR SIMULATIONS

For technical reasons, direct correlations of individual cell WI with lag-time were not possible in our experiments. However, measuring the lag-times of individual cells on agarose using a high numerical aperture objective in an independent experiment, we could determine the lag-time distribution for different growth conditions prior to agarose seeding (Fig. 4 in main text). We then used these lag-time distributions in our simulations - *i.e.* in the simulations, we assigned lag-times to individual founder cells by picking from the experimentally determined lag-time distribution. In the simulations, we can track the fate of individual cells and so can correlate the eventual WI with each lag-time.

Fig. 5 and Fig. 6 show the results of this analysis for both the exponential, and heat-shocked lag-time cell distributions for different initial cell densities, and for different growth scenarios (*i.e.*, perimeter only growth, and exponential area growth, see main text). The winner index is inversely correlated with lagtime in all cases, with the strongest correlation seen in the low density, exponentially expanding colonies.

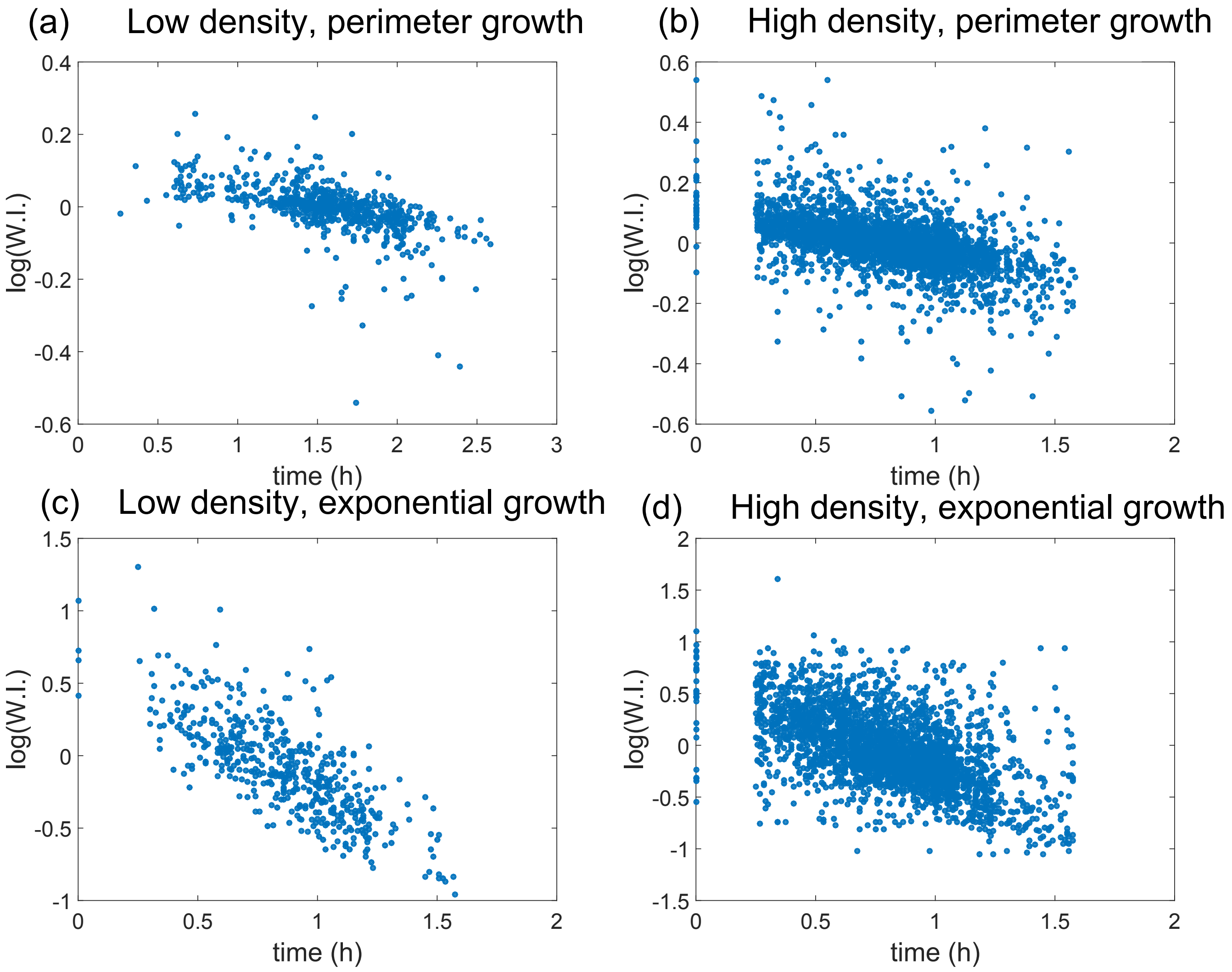

Figure 5: log(WI) versus lagtime for individual cells in our simulations, where the lagtimes assigned to each cell have been picked from an experimentally determined distribution. The lag-times assigned in this case are ones measured in cells which had been picked from liquid culture while still in the exponential phase, before being spread on an agarose surface (Fig. 4a of the main text). N is the number of initial cells scattered over a 500x500 square lattice. (a)Low density ($N = 100$), perimeter growth, (b) High density ($N = 400$), perimeter growth, (c) Low density, exponential growth (d) High density, exponential growth.

---

[1] Grant, M., Waclaw, B., Allen, R. J. & Cicuta, P. 2014 The role of mechanical forces in the planar-to-bulk transition in growing *escherichia coli* microcolonies. *J. R. Soc. Interface*, **11**. (doi:10.1098/rsif.2014.0400).

V. MULTI DIMENSIONAL SCALING

Multi Dimensional Scaling (MDS) was used to assess the similarity between Fourier spectra describing the shapes of colonies and Voronoi polygons. This technique allows for a visual assessment of the similarity between a group

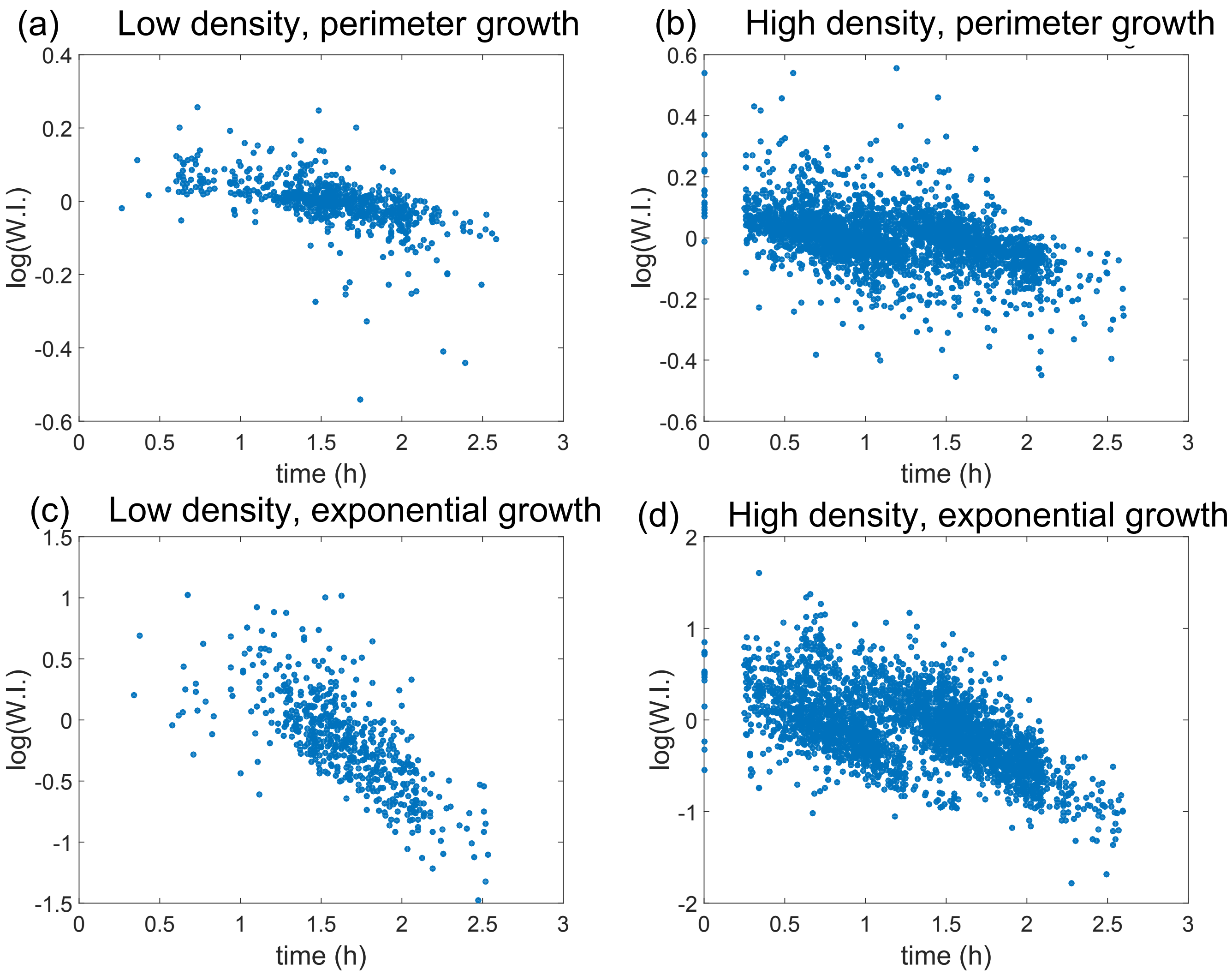

Figure 6: log(WI) versus lagtime for individual cells in our simulations, where the lagtimes assigned to each cell have been picked from an experimentally determined distribution. The lag-times assigned in this case are ones measured in cells which had been heat shocked for 15 min at 50 °C while in the stationary phase, before being spread on an agarose surface (Fig. 4b of the main text). N is the number of initial cells scattered over a 500x500 square lattice. (a) Low density ($N = 100$), perimeter growth, (b) High density ($N = 400$), perimeter growth, (c) Low density, exponential growth (d) High density, exponential growth.

of objects, as defined by a similarity function, where each object is represented as a single point. In the simplest terms, objects which are more similar to each other will be plotted on an N-dimensional plot closer together than those which are dissimilar. Clearly, a three-dimensional plot is the highest N which might be visualised, and more generally, two dimensions is chosen, since it's the easiest and most intuitive to understand. However, reducing the dimensionality of the resulting plot introduces more *stress* into the resulting object map, since it becomes harder to reflect the true inter-object similarity distances with fewer dimensions. This stress can be quantified to help assess the MDS plot's fidelity in representing the 'true' inter-object similarity. Intuitively, the lower the stress metric, the better the plot captures the inter-object similarity. Below, we detail exactly how the similarities and stress metric were calculated.

A. Calculating Fourier spectra similarities and stress

The similarities between each Fourier spectra were calculated as the total pairwise Euclidean distances between each of the respective Fourier descriptor terms, f_{n},$-5 \leq n \leq 5$, describing each shape:

$$s(x_{ij}) = \sqrt{\left(\sum_{n=-5}^{n=5} (f_{i_n} - f_{j_n})^2 \right)} \tag{1}$$

where $s(x_{ij})$ is the Euclidean distance between the i'th and j'th Fourier spectra (our objects). Using this as our measure of similarity between each Fourier spectra we wished to compare, we performed MDS analysis using MATLAB's `cmdscale` function.

The stress was then computed using the Kruskal Stress metric, defined as:

$$K = \sqrt{\frac{\sum \sum (s(x_{ij}) - d_{ij})^2}{\sum d_{ij}^2}} \tag{2}$$

where d_{ij} is the resulting projected distance between the the i'th and j'th objects on the MDS map. Clearly, the more distortion introduced by reducing the dimensionality of the MDS plot increases K.
